# Stricture of the Urethra

**Published:** 1876-11

**Authors:** B. F. Maury

**Affiliations:** Philadelphia


### STRICTURE OF THE URETHRA.

Clinic, By B. F. MAURY, M.D., Philadelphia.

This man, aged thirty-five, comes before us with some urinary difficulty, which we suspect to be a stricture of the urethra. The first thing I will do will be to have him lie down between blankets, so that he shall be kept as warm as possible, with his shoulders elevated by pillows, and his knees drawn up. Such a position is the easiest in which to explore the canal, and the best to observe, until experience shall warrant your modifying it at your own discretion. His body is covered with a blanket, and another protects his legs, and between these I can get at the region about the penis.

Now, in reply to questions I ask, he tells us that he has no water in his bladder; that he made water about ten minutes ago, voluntarily, and not on account of nervousness about coming into the clinic; and that he also made water about a half hour prior to that. This point of nervousness you must never lose sight of, for it is important. Many a man cannot urinate if he knows any one is looking at him, and you are aware how irritable are the bladders of medical students when about to be examined. So don't forget to make allowance for the influence it may have upon the action of a patient's bladder.

The man tells us, also, that he passes water about once every hour during the day, and once every half hour at night. So he does it about thirty-six times in twenty-four hours. I need scarcely say this is much too often. He should never feel a strain upon it, or be painfully aware of its existence. In the normal condition, the mucous membrane tolerates healthy urine in moderate quantity without any sensation; but if the urine cannot be freely voided, or if from any cause it be retained long enough for the salts to be precipitated, the bladder resists and becomes irritable. Then it will act often and with undue violence. Such is the result of stricture of the urethra; and then usually follows hypertrophy of the muscular coat, just as in a blacksmith the biceps is excessively developed by excessive use. An enlarged prostate may produce the same effect; but you would scarcely expect such a cause in a man of thirty-five. A calculus might do the same, but there is none in this case.

Now I will explore the urethra. I tell the man to breath through his mouth, so as to prevent his straining; and, taking a Sir Henry Thompson's bougie, No. 20, well oiled and moderately warmed, I insinuate it into the meatus urinarius. Not that I expect to pass it into the bladder, but because a large instrument should be used for the first exploration. The instrument glides gently downward for about an inch, and then meets a slight obstruction. Apply the least possible pressure, it slips through, and I feel something tear. Observe, I used no force. My rule is, if no good is done, at least to do no harm. You might ask if I am not afraid to make the little laceration which I have made, the evidence of which is seen in these few drops of blood? I say, "No!" If it were low down, below the spongy portion, I should be afraid of making a false passage; but not here. As it is, I have not given any pain, nor used any undue violence. There is no need to use ether; and I rarely have recourse to it. Acting gently, kindly and delicately will save you many an embarrassment, and secure the confidence of your patients. Now I find another constriction which resists the gentle pressure I make, so I take a No. 17, which passes through to the bladder. I know it has safely arrived, because the handle takes a position with its flat surfaces looking directly upward and downward, and I find the curved part can be freely swept round in the bladder. This motion could not be possible if the instrument were anywhere else.

At this point I complete my examination of the patient by inserting my finger into the rectum. In doing this, always see that your finger has no hang-nails or sores upon it, and that it is well oiled, because it would be easy to contract syphilis, if it existed in the patient, were these precautions neglected, not only in hospitals, but also in private practice.

I coax the finger in with a gentle rotatory motion, and find, to my surprise, a very much enlarged prostate. This is quite uncommon in a man so young, and proves the importance of not neglecting to be thorough.

I now take larger instruments, and pass successively No. 18, No. 19, and No. 20. The last is the one which failed in the first instance; but the stricture has been gradually dilated, until it passes with little difficulty. This process of "gradual dilatation" is the safest, easiest and most readily accomplished by young practitioners. It is the one I would recommend you to use, remembering always that every operation upon the urethra, however simple, may give rise to a fatal result. The simple passing of a sound has been followed by death, at the hands of some of the most eminent surgeons that ever lived. Therefore, you must always use the utmost caution, seeing that your patients are in as good condition as possible, that they suffer no unnecessary exposure, and that each act of your own is undertaken with the greatest care.

For our patient, I shall order three grains of quiniæ sulph. ter die, the avoidance of all stimulants, and the use of large diluent drinks. He shall receive a quart of barley-water daily, to each tumblerful of which a drachm and a half of spirits ætheris nitrosi shall be added. Less than this quantity, I think, does no good. Nor would I use any stimulating or mineral diuretic.

Because he has an enlarged prostate gland, I will order a warm bath daily, and, if necessary, a half dozen leeches to the perineum. The use of sounds will be renewed after a few days, and continued for some time, with gradually increasing intervals, after the urethra has been dilated to such a calibre as shall seem expedient. When he goes away from us he will be given an appropriate instrument, and taught how to use it, so that he may prevent the recurrence of the stricture. In this way only can he be sure to avoid having the same trouble he has now.—*Med. and Surg. Reporter*.

